# The Effector AGLIP1 in *Rhizoctonia solani* AG1 IA Triggers Cell Death in Plants and Promotes Disease Development Through Inhibiting PAMP-Triggered Immunity in *Arabidopsis thaliana*

**DOI:** 10.3389/fmicb.2019.02228

**Published:** 2019-09-26

**Authors:** Shuai Li, Xunwen Peng, Yingling Wang, Kangyu Hua, Fan Xing, Yuanyuan Zheng, Wei Liu, Wenxian Sun, Songhong Wei

**Affiliations:** ^1^Department of Plant Pathology, College of Plant Protection, Shenyang Agricultural University, Shenyang, China; ^2^Department of Chemistry & Biochemistry, The Ohio State University, Columbus, OH, United States; ^3^College of Plant Protection, Jilin Agricultural University, Changchun, China

**Keywords:** *Rhizoctonia solani*, effector, innate immunity, defense responses, fungal virulence and pathogenicity

## Abstract

*Rhizoctonia solani*, one of the most detrimental necrotrophic pathogens, causes rice sheath blight and poses a severe threat to production. Focus on the function of effectors secreted by necrotrophic pathogens during infection has grown rapidly in recent years. However, little is known about the virulence and mechanisms of these proteins. In this study, we performed functional studies on putative effectors in *R. solani* and revealed that AGLIP1 out of 13 putative effectors induced cell death in *Nicotiana benthamiana*. AGLIP1 was also demonstrated to trigger cell death in rice protoplasts. The predicted lipase active sites and signal peptide (SP) of this protein were required for the cell death-inducing ability. *AGLIP1* was greatly induced during *R. solani* infection in rice sheath. The AGLIP1’s virulence function was further demonstrated by transgenic technology. The pathogenesis-related genes induced by pathogen-associated molecular pattern and bacteria were remarkably inhibited in AGLIP1-expressing transgenic *Arabidopsis* lines. Ectopic expression of AGLIP1 strongly facilitated disease progression in *Arabidopsis* caused by the type III secretion system-defective mutant from *Pseudomonas syringae* pv. tomato DC3000. Collectively, these results indicate that AGLIP1 is a possible effector that plays a significant role in pathogen virulence through inhibiting basal defenses and promoting disease development in plants.

## Introduction

*Rhizoctonia solani* (teleomorph: *Thanatephorus cucumeris*) is classified as a saprophytic fungus, which resides in the soil in the form of sclerotia and does not produce asexual spores. It is complex, with more than 100 species which infect crops, such as rice, wheat, corn, cotton and soybean, ornamental, and horticultural plants. *R. solani* is divided into 14 anastomosis groups (AG1 to AG13 and AGBI). Based on differences in culture characters, host, physiology and biochemistry, they are divided into different subgroups (Ogoshi, 1987; Anderson et al., 2017). Among them, AG1 IA is the most destructive group of pathogens that causes diseases in many monocot and dicot plants. The second most serious rice disease, rice sheath blight, which can reduce rice production up to 50%, is also brought on by AG1 IA (Bernardes-de-Assis et al., 2009).

Pathogenic mechanisms are significantly different among various types of pathogens which allows for characterization of plant pathogens into biotrophic, hemibiotrophic, and necrotrophic pathogens according to their life styles. Biotrophic pathogens obtain nutrients from host living cells and tissues by manipulating host physiology, while hemibiotrophic pathogens absorb nutrients from living cells in the early biotrophic stage of infection, and then obtain nutrients by killing host cells in the later necrotrophic stage of infection (Schulmeyer and Yahr, 2017). Usually, biotrophic and hemibiotrophic pathogens secrete effectors to facilitate infection by manipulating the structure and function of the host cells and suppressing plant defenses. Effectors that are secreted and transported into host cells play important roles in pathogenicity of biotrophic and hemibiotrophic fungi (Koeck et al., 2011). Necrotrophic pathogens, such as *R. solani*, have long been known as plant killers. Necrotrophic fungi complete their life cycle by killing host cells and take nutrients from dead plant tissue. Such pathogens secrete large amounts of cell wall degrading enzymes or toxins, which promote cell necrosis for their own development (Oliver and Solomon, 2010).

However, recent studies indicate that the infection process of necrotrophic pathogens is complex. There may be a transient transition from biotrophy to necrotrophy in the life cycle of such pathogens (Kabbage et al., 2015). For example, *Botrytis cinerea* produces an exopolysaccharide, which regulates the antagonistic effects of jasmonic (JA) and salicylic acid (SA) signaling pathways to enhance its pathogenicity in tomato (El et al., 2011). Moreover, effectors are also crucial weapons which play important roles in promoting pathogen infection. SSITL secreted by *Sclerotinia sclerotiorum* is a possible effector that inhibits host resistance mediated by the JA/ethylene (ET) signaling pathway during the early stage of pathogen infection (Zhu et al., 2013). SsCP1, A cerato-platanin protein, which targets pathogenesis-related protein 1 (PR1), regulates the concentration of SA and contributes to the virulence of *S. sclerotiorum* (Yang et al., 2018). Interestingly, it has recently been shown that NIS1, a core effector in *Colletotrichum* spp. interacts with pattern recognition receptor (PRR)-associated kinases BAK1 and BIK1. Such interaction inhibits kinase activities and the BIK1-NADPH oxidase interaction in host plants (Irieda et al., 2019). *C. orbiculare* expresses specific effectors at different stages. *C. orbiculare* accumulate virulence-related effectors in a pathogen-host interface during the early biotrophic phase and are secreted into plant cells. This process is regulated by the Rab GTPase SEC4 protein (Irieda et al., 2014). The *Parastagonospora nodorum* effectors SnToxA and SnTox3 interact with PR-1-5 and PR-1-1, respectively, and play a decisive role in pathogenicity (Lu et al., 2014; Breen et al., 2016). SnTox1 secreted by *P. nodorum* is a dual-function protein that facilitates infection and counters wheat-produced chitinases (Liu Z. et al., 2016).

Some plants initiate innate immunity through specific interactions of pathogen effectors by nucleotide binding-leucine rich repeat (NB-LRR) proteins. The recognition usually leads to plant cell death, also known as hypersensitive responses (HRs). For biotrophic and hemibiotrophic fungi, HRs is an obstacle for further development in early infection stages (Stergiopoulos and de Wit, 2009). However, host cell death may be beneficial rather than detrimental for necrotrophic pathogenesis. Effectors in necrotrophic fungi may facilitate host cell wall degradation and ultimately promote infection (McDonald and Solomon, 2018). For example, a small protein SsSSVP1 in *S. sclerotiorum* interacts with QCR8, a subunit of the cytochrome b-c1 complex of the mitochondrial respiratory chain in plants. This interaction results in significant plant cell death and facilitates pathogen infection (Lyu et al., 2016). Furthermore, Ss-Caf1 and Xyn11A secreted by *S. sclerotiorum* and *B. cinerea*, respectively, may interact with specific host proteins or unknown substances in host cells which, subsequently, could result in host cell death and contribute to pathogenesis (Noda et al., 2010; Xiao et al., 2014). In *P. tritici-repentis*, the effector proteins ToxA interacts with *Tsn1*, a dominant wheat susceptibility gene, while effector protein ToxB interacts with *TscB* in a gene-for-gene relationship to cause chlorosis in susceptible wheat lines (Sperschneider et al., 2017).

*Rhizoctonia solani* encodes multiple secreted proteins which are considered as effectors, some of which cause necrotic phenotypes in rice, corn, and soybean (Zheng et al., 2013). However, whether effectors in the necrotrophic pathogen can trigger defense signaling after being recognized by host and non-host plants is still unclear. In this study, we investigated 13 putative effectors in *R. solani* and their ability to induce cell death through transient expression assays. An effector named AGLIP1 (Gene ID: AG1IA_05142) was discovered to trigger cell death in *Nicotiana benthamiana* and rice protoplasts, respectively. The secretion signal peptide (SP) and predicted lipase active sites of AGLIP1 were found to play an important role in inducing cell death. Importantly, our findings also demonstrated that heterologous expression of AGLIP1 in transgenic *Arabidopsis* plants promotes bacterial pathogens progression through suppressing defense responses, which includes flg22- and chitin-triggered PR genes expression. The findings will provide new perspectives in understanding the molecular mechanisms of *R. solani* pathogenesis.

## Results

### A Putative Effector AGLIP1 in *R. solani* Induces Cell Death in *N. benthamiana*

**FIGURE 1:**
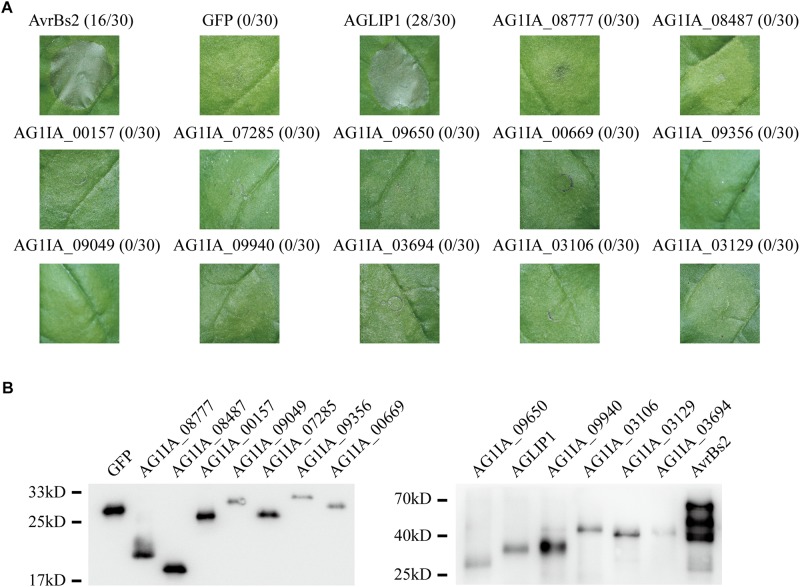
Putative effectors in *Rhizoctonia solani* induce cell death symptoms on *Nicotiana benthamiana* leaves. **(A)** One protein out of 13 tested putative effectors, i.e., AGLIP1, induced the cell death in *N. benthamiana*, while the other 12 proteins did not. GFP, which did not trigger cell death, was shown as a negative control. AvrBs2 in *X. oryzae* pv. *oryzicola* was shown as a positive control. Numbers, e.g., 16/30, indicate that 16 of 30 infiltrated leaves exhibited cell death phenotypes. Representative photos were taken 3 days after DEX treatment. **(B)** Transient expressions of 13 tested putative effectors, AvrBs2 and GFP in *N. benthamiana* were confirmed by Western blot assay. For AGLIP1, the sample for protein extraction was collected at 1 day after DEX treatment, while samples for other proteins extraction were collected at 3 days after DEX treatment. The proteins with 3 × HA tag were detected by immunoblotting with an anti-HA antibody.

Many effectors in different pathogens induce non-host hypersensitive cell death in *N. benthamiana* (Li et al., 2015; Fang et al., 2016). To identify if any effectors in *R. solani* have the ability to induce cell death, we chose 13 putative effectors specifically that contained conserved domain and predicted functions (Supplementary Table S1). 13 putative effectors were transiently expressed in *N. benthamiana* through *Agrobacterium tumefaciens*-mediated transfection after amplifying and subcloning into the glucocorticoid-inducible pTA7001 binary vector (Aoyama and Chua, 1997). The cell death symptoms were recorded within 3 days post-treatment with dexamethasone (DEX), which induces expression of effectors in *N. benthamiana*. Among the 13 tested effectors, AGLIP1 was shown to trigger cell death in *N. benthamiana* leaves at 1–2 days after DEX spraying, while the expression of green fluorescent protein (GFP) did not induce necrosis in *N. benthamiana*. AvrBs2 in *Xanthomonas oryzae* pv. *oryzicola* was shown as a positive control (Li et al., 2015). Other investigated putative effectors did not induce cell necrosis in *N. benthamiana*, although expressions of those proteins were all detected by Western blotting (Figures 1A,B).

To discriminate whether the induction of cell death is caused by the hypersensitive response triggered by activation of the resistance gene following recognition of effector presence, or the possible toxicity of AGLIP1 in the plant cell, two effector-triggered immunity (ETI) marker genes, *NbPR1*, and *NbHsr203J* (Wei et al., 2013) were detected by quantitative real time reverse transcription-polymerase chain reaction (qRT-PCR) after 1 day induced-expression of AGLIP1 and control protein GFP in *N. benthamiana*. However, expression of the two genes showed no significant differences compared with DEX-induced expression of AGLIP1 and mock treatment (Supplementary Figure S1). This result indicated that AGLIP1-triggered cell death might not result from ETI but rather from cellular toxicity.

### AGLIP1 Is Highly Conserved in Plant Fungal Pathogens

AGLIP1 encodes a 302 amino acid protein which contains a predicted N-terminal SP and a C-terminal lipase domain (Supplementary Figure S2A). Previous study demonstrates that core effector proteins are highly conserved among many pathogenic fungi (Lyu et al., 2016). BLAST searches against the NCBI database found that the lipase domain proteins appeared in many fungi and bacterial. Phylogenetic analysis indicated that homolog of AGLIP1 were widely present in plant pathogenic fungi, in particular necrotrophic pathogens (Supplementary Figure S2B). In order to investigate sequence conservation of these homologous proteins, we performed multiple amino acid alignment analysis, which showed AGLIP1 is highly conserved, and similar to known plant fungal pathogens proteins (Supplementary Figure S2C).

### The Predicted Lipase Active Sites and Signal Peptide of AGLIP1 Are Required for Its Ability of Cell Death-Eliciting

**FIGURE 2:**
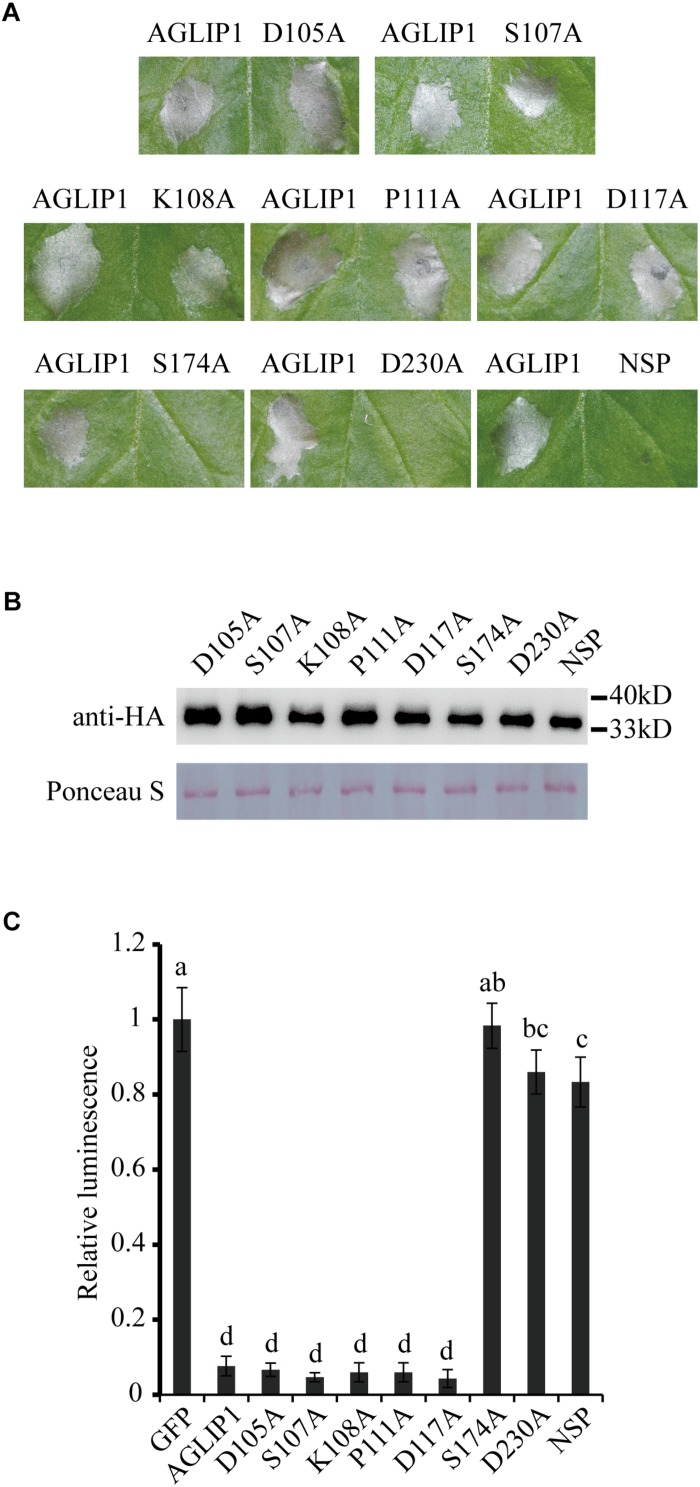
The predicted lipase active sites and signal peptide of AGLIP1 are required for its cell death-inducing ability. **(A)** The mutant proteins AGLIP1^S174A^, AGLIP1^D230A^ and AGLIP1^NSP^ lost the ability to induce cell death, while other variants, including AGLIP1^D105A^, AGLIP1^S107A^, AGLIP1^K108A^, AGLIP1^P111A^ and AGLIP1^D117A^, triggered cell death symptoms on *N. benthamiana* leaves. **(B)** The protein expression level of mutant proteins in the infiltrated leaves detected by Western blotting. The equal loading of the total proteins was showed by Ponceau S staining. The samples for protein extraction were collected before the cell death symptoms were visible. The proteins with 3 × HA tag were detected by immunoblotting with an anti-HA antibody. **(C)** The luciferase activity in rice protoplasts was significantly inhibited by the co-expression of AGLIP and its mutant variants AGLIP1^D105A^, AGLIP1^S107A^, AGLIP1^K108A^, AGLIP1^P111A^ and AGLIP1^D117A^, while the mutant variants AGLIP1^S174A^, AGLIP1^D230A^, and AGLIP1^NSP^ did not. Data are means ± standard error (SE). Different letters (a through d) indicate significant differences in the luciferase activity at *P* < 0.05, according to Duncan’s multiple-range test.

To test the function of AGLIP1’s lipase activity and SP in cell death induction, point and deletion mutations were constructed, respectively. The residues Asp105, Ser107, Lys108, Pro111, Asp117, Ser174, and Asp230 which were predicted as active sites of the conserved lipase domain were substituted with alanine (Supplementary Figure S2A). Cell death symptoms on *N. benthamiana* leaves were monitored within 3 days after infiltration of *Agrobacterium* containing the AGLIP1 mutation and DEX treatment. Interestingly, expression of AGLIP1^S174A^, AGLIP1^D230A^, and the truncated variant without signal peptide (NSP) did not cause cell necrosis, while expression of other variants also caused cell death symptoms in *N. benthamiana* (Figure 2A). Western blot analysis showed that all of the different AGLIP1 mutations were expressed at similar levels in the infiltrated leaves (Figure 2B).

To further verify whether AGLIP1 and its variants induce cell death in host, we utilized a polyethylene glycol (PEG)-mediated transfection system for transiently expressing these proteins in rice protoplasts (Chen et al., 2013). The recombinant plasmids containing the full-length sequence of *AGLIP1* and its variants were co-transformed into rice protoplasts with luciferase (LUC) protein driven by *Cauliflower mosaic virus* 35S promoter, respectively (Luehrsen et al., 1992). LUC activity was tested for identifying the ability of cell death-inducing in rice protoplasts which is isolated from rice cv. Nipponbare. As compared with the LUC intensity in the protoplasts which were co-transfected with GFP, LUC activities were significantly low when AGLIP1, AGLIP1^D105A^, AGLIP1^S107A^, AGLIP1^K108A^, AGLIP1^P111A^, and AGLIP1^D117A^ were co-expressed with LUC, respectively. By contrast, LUC activity did not have any inhibitory effect in rice protoplasts which expressed AGLIP1^S174A^, AGLIP1^D230A^, and AGLIP1^NSP^ (Figure 2C). These results demonstrated that AGLIP1 could trigger cell death and the putative lipase active sites S174, D230, and the protein’s SPs are indispensable for its ability to elicit plant cell death.

### *AGLIP1* Is Up-Regulated During *R. solani* Infection and Located at Endoplasmic Reticulum (ER)

**FIGURE 3:**
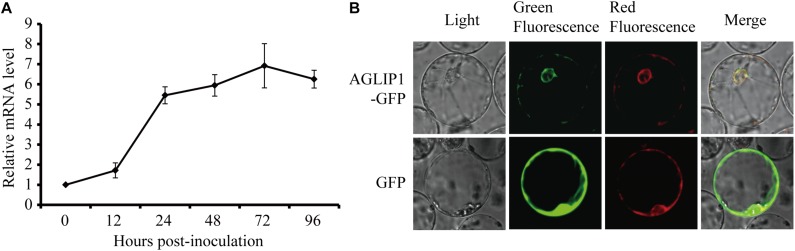
Expression analyses of *AGLIP1* during *R. solani* infection and subcellular localization of AGLIP1 in rice cell. **(A)** Expression analyses of *AGLIP1* during *R. solani* infection in rice cv. Nipponbare. The *R. solani*-inoculated rice sheaths were collected at 0, 12, 24, 48, 72, and 96 h post-inoculation for gene expression analyses using quantitative real time reverse transcription-polymerase chain reaction assay. The expression level of *gpd* was used as an internal reference for normalizing within the samples. Data are means ± standard error. **(B)** Subcellular localization of AGLIP1-GFP transiently expressed in rice protoplasts. The vector pUC19 carrying GFP was used as a control. The overlapped fluorescence was observed in rice protoplasts when co-expressed with AGLIP1-GFP and HDEL-mCherry via laser scanning confocal microscopy. The photo was taken before cell death symptom was induced.

The effector genes are often up-regulated during filamentous plant pathogen infection (Stergiopoulos and de Wit, 2009). To investigate regulation of *AGLIP1* expression during *R. solani* infection, the strain collected from a heavily infected rice plant in Liaoning province, China, was artificially inoculated into rice sheath. *AGLIP1* expression at 0, 12, 24, 48, 72, and 96 h post-inoculation was measured via qRT-PCR. The result showed that *AGLIP1* expression was transcriptionally induced from approximately 2- to 8-fold at different times during infection (Figure 3A). This result demonstrated that *AGLIP1* expression was regulated during *R. solani* infection and indicated that AGLIP1 might have essential functions in the interaction between rice and the fungal pathogen.

To investigate subcellular localization of AGLIP1 in rice cells, *AGLIP1* coding sequence was amplified, and fused in frame with the GFP gene at its C terminus, then subcloned into the pUC19 plasmid driven by 35S promoter. The recombinant AGLIP1-GFP protein was transiently co-expressed in rice protoplasts with the known ER marker HDEL-mCherry (Haseloff et al., 1997). The result showed that green fluorescence from AGLIP1-GFP and red fluorescence from HDEL-mCherry overlapped, suggesting AGLIP1 is ER located (Figure 3B). The similar subcellular localization pattern of AGLIP1-GFP has also been observed in *N. benthamiana* (Supplementary Figure S3). Induced expression of AGLIP1-GFP in *N. benthamiana* could also trigger cell death, demonstrating the AGLIP1-GFP fusion protein is functional (data not shown).

### Ectopic Expression of AGLIP1 Suppresses PTI Signaling in *Arabidopsis thaliana* Seedlings

To investigate if AGLIP1 suppresses plant immunity, we generated *AGLIP1* transgenic *Arabidopsis* lines through *Agrobacterium*-mediated transformation. In 7 transgenic overexpression lines, AGLIP1 expression was driven by a DEX-inducible promoter. Expressions of AGLIP1 in these transgenic lines were detected by immunoblotting (Supplementary Figure S4). Three independent T3 homozygous overexpression lines, Line 3, Line 4 and Line 5, were chosen for subsequent functional analyses.

**FIGURE 4:**
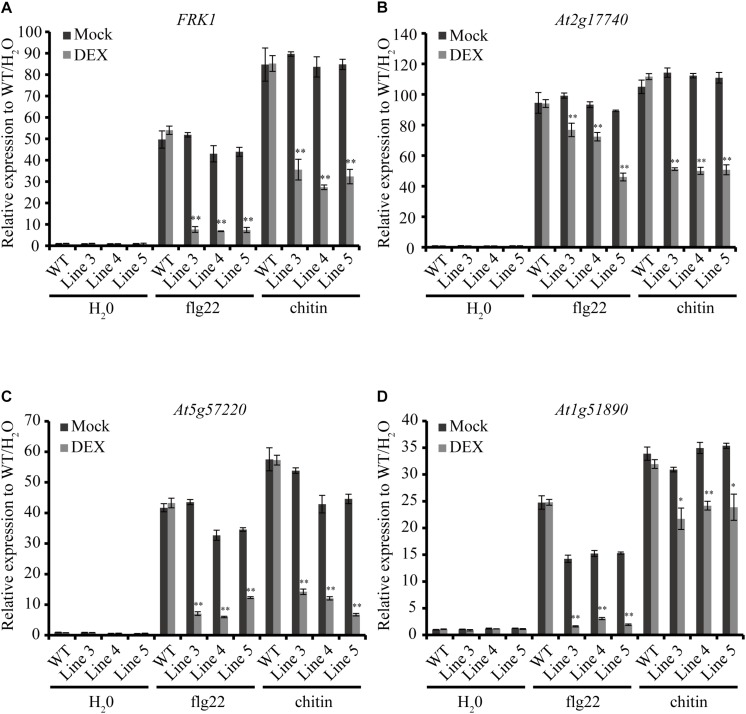
Heterologous expression of AGLIP1 suppresses PAMP-induced defense genes expression in transgenic *Arabidopsis thaliana* seedlings. **(A–D)** Upregulation of the defense marker genes *FRK1*, *At2g17740*, *At5g57220*, and *At1g51890*, respectively, induced by flg22 and chitin were dramatically suppressed in transgenic Line 3, Line 4, and Line 5 after DEX-induced expression of AGLIP1. The transgenic plant seedlings were treated with 10 μM DEX or 0.03% ethanol as mock control for 24 h, followed by the treatment of 1 μM flg22 or chitin for 3 h. The expression level of *AtUBQ5* was used as an internal reference for normalizing within the samples. Asterisks (^∗^) indicate *P* value < 0.05 and (^∗∗^) indicate *P* value < 0.01; means ± standard error are shown.

As an important weapon, effectors secreted by pathogens usually suppress plant defense responses including *PR* genes expression (Boller and He, 2009). Here, we chose four early defense-response genes, *FRK1* (Flg22-induced receptor-like kinase 1), *At2g17740* (cysteine/histidine-rich C1 domain family protein), *At5g57220* (member of CYP81F) and *At1g51890* (leucine-rich repeat protein kinase), which can be induced by pathogen-associated molecular patterns (PAMPs) such as bacterial flagellin and fungal chitin but not by stress-related signals (He et al., 2006; Akimoto-Tomiyama et al., 2012). Expression patterns of the four genes were detected by qRT-PCR in the *AGLIP1* transgenic plants after DEX treatment followed by flg22 and chitin stimulation. Remarkably, expression of the four genes induced by flg22 and chitin were dramatically suppressed in all transgenic lines after DEX-induced expression of AGLIP1 (Figures 4A–D). These results indicated that AGLIP1 contributes to virulence by inhibiting PAMP-triggered immune signaling in plants.

### Ectopic Expression of AGLIP1 Promotes Disease Development via Suppressing PTI Responses in *Arabidopsis* Plants

**FIGURE 5:**
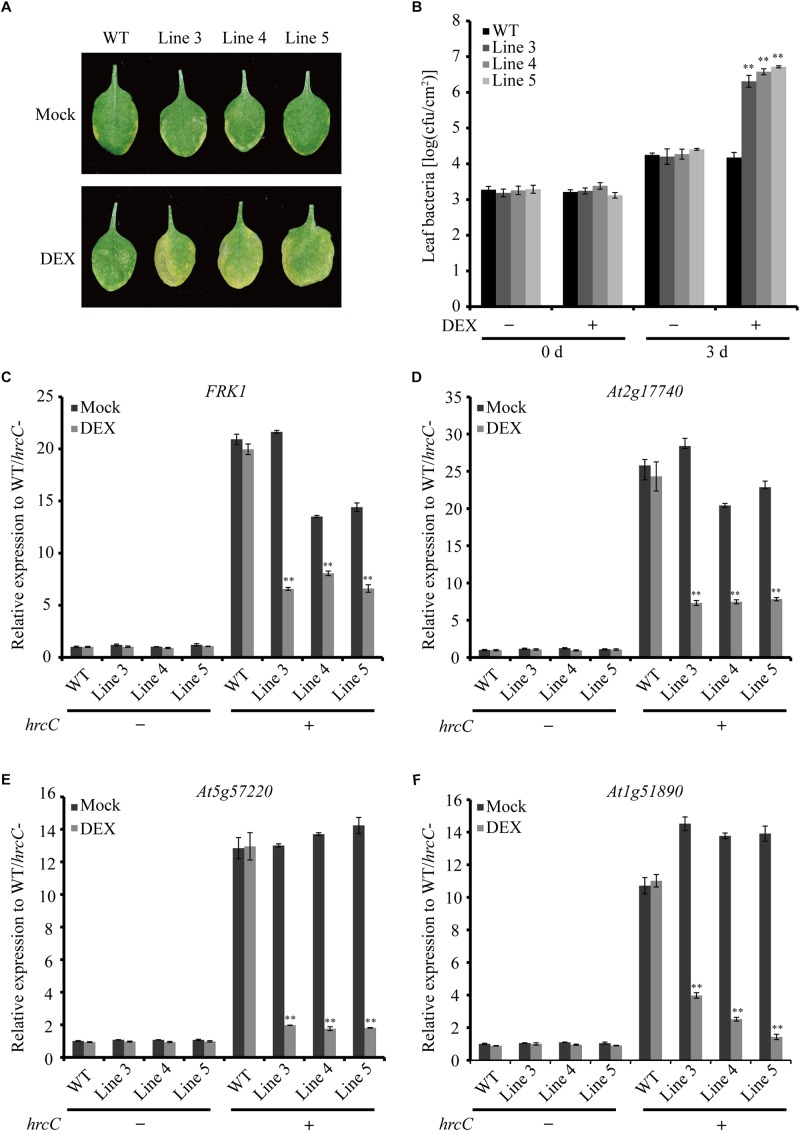
Heterologous expression of AGLIP1 suppresses PTI signaling and promotes disease development in transgenic *Arabidopsis* plants. **(A)** Disease symptoms in the wild-type and AGLIP1 transgenic *Arabidopsis* plant lines after inoculation with bacterial pathogens *Pseudomonas syringae* pv. tomato (*Pst*) DC3000 *hrcC* mutant. Disease symptoms exhibited on the leaves of the wild-type and *AGLIP1* transgenic lines Line 3, Line 4 and Line 5 with mock or DEX treatment after pressure infiltration with the *Pst* DC3000 *hrcC* mutant. Photos were taken at 3 days after inoculation. **(B)** *In planta* bacterial population of *Pst* DC3000 *hrcC* mutant in the wild-type and *AGLIP1* transgenic lines at 0 day and 3 days after inoculation. **(C–F)** Upregulation of the defense marker genes *FRK1*, *At2g17740*, *At5g57220*, and *At1g51890*, respectively, induced by *Pst* DC3000 *hrcC* mutant were completely inhibited in transgenic Line 3, Line 4 and Line 5 after DEX-induced expression of AGLIP1. The 4–5 weeks transgenic plant were treated with 30 μM DEX or 0.1% ethanol as mock control for 24 h, followed by the spray inoculation of *Pst* DC3000 *hrcC* mutant for 6 h. The expression level of *AtUBQ5* was used as an internal reference for normalizing within the samples. Asterisks (^∗∗^) indicate *P* value < 0.01; means ± standard error are shown.

To verify the virulence function of AGLIP1 in suppressing plant immunity further, the AGLIP1-expressing transgenic *Arabidopsis* plants were first inoculated with the *Pseudomonas syringae* pv. tomato (*Pst*) DC3000 *hrcC* mutant that is defective in the type III secretion system (T3SS) apparatus via pressure infiltration (He et al., 2006; Hatsugai et al., 2018). The results indicated increased levels of disease symptoms on inoculated transgenic leaves after DEX treatment compared to plants under mock spraying (Figure 5A). The leaf bacterial growth assay proved that the population of *Pst* DC3000 *hrcC* mutant in the transgenic plants with DEX treatment was also remarkably enhanced compared to the mock-treated or wild-type transgenic plants at 3 days post-inoculation. Furthermore, the bacterial population in the transgenic Line 5 was higher than the other two lines, Line 3, and Line 4. These results are consistent with the higher AGLIP1 expression level in Line 5 after DEX induction (Supplementary Figure S4). Moreover, the bacterial population in the wild-type plants was similar after DEX and mock treatments, indicating the lack of influence that DEX has on proliferation of the bacterium (Figure 5B). In addition, we showed that the expression of *FRK1*, *At2g17740*, *At5g57220*, and *At1g51890* induced by *Pst* DC3000 *hrcC* mutant in transgenic lines with mock treatment was markedly inhibited by DEX-induced AGLIP1 expression, which was consistent with the results obtained from transgenic *Arabidopsis* seedlings (Figures 5C–F). Taken together, these findings demonstrated that AGLIP1 expressed in transgenic *Arabidopsis* plants facilitated bacterial multiplication and the development of disease symptom through inhibiting plant basal defenses during pathogen infection.

## Discussion

Rice, one of the major food crops, is continuously threatened by various pathogenic microbes. Pressingly, *R. solani* causes rice sheath blight, one of the most severe fungi diseases in rice, and poses a significant threat to grain yield (Shu et al., 2019). Breeding for disease-resistant varieties is considered to be the most effective and eco-friendly method for disease control. However, no endogenous resistance gene for rice sheath blight has been identified in rice besides a selection of moderately resistant rice varieties (Zheng et al., 2019). *R. solani* has been described as a saprophyte that takes nutrients from dying plant debris/cells to complete their life cycle. Recent studies have shown that the effector proteins secreted by necrotrophic pathogens mainly designed as host-specific or host-selective toxins are able to promote necrosis and play important roles in the host-pathogen interactions (Lyu et al., 2016; Anderson et al., 2017). Almost 900 secreted proteins are predicted in the *R. solani* genome, many of which are thought to be candidates of effector proteins. The genes of many putative effectors are up-regulated during rice infection via expression profiling analyses, indicating effectors may play significant roles in the interaction between rice and the pathogen (Zheng et al., 2013; Anderson et al., 2017).

In this study, a heterogeneous transient expression assay was used to investigate the *R. solani* putative effectors activity of cell death-eliciting in the non-host *N. benthamiana* plants and revealed that one of them, i.e., AGLIP1, caused cell death (Figure 1). Furthermore, AGLIP1 was found to possess the function of eliciting cell death in host rice protoplasts (Figure 2C). Similar results were found in putative effectors from *R. solani* AG1 IA and AG8 strains which induce cell death symptoms in rice and non-host *N. benthamiana*, respectively (Zheng et al., 2013; Anderson et al., 2017). Through Pfam and BLAST searches against the NCBI database, AGLIP1 was predicted to have a lipase domain. Effector proteins which contain the lipase domain have been reported in regulating innate immunity in humans and in plants (Blumke et al., 2014; Chen and Alonzo, 2019). However, studies on induction of cell death by fungal effectors with the lipase domain have not been reported so far. We predicted 7 enzymatically active sites in the protein and 2 of them, i.e., S174 and D230, were essential for inducing cell death both in *N. benthamiana* and in rice protoplasts (Figure 2). Extended results showed that the expression level of two ETI marker gene, *NbPR1* and *NbHsr203J*, were not different between DEX-induced expression of AGLIP1 and mock treatment leaf tissue, demonstrating the cell death was likely toxin-induced necrosis rather than active hypersensitive response triggered by the activation of a resistance gene (Supplementary Figure S1). These findings indicate that lipase domain activity of AGLIP1 is essential for its cell death-inducing activity in plants.

The SP of AGLIP1 was also required for its cell death-inducing ability in *N. benthamiana* and in rice protoplasts (Figure 2). Similar results have been reported that the full-length effectors MoCDIPs in *Magnaporthe oryzae* and UV_44 in *Ustilaginoidea virens* are able to trigger cell death in rice protoplasts, respectively, but truncated versions of these proteins without SPs do not (Chen et al., 2013; Fang et al., 2016). The function of SP for secreted proteins in inducing cell death suggests that these effector proteins are likely to function in plant intercellular space. Effectors without SPs cannot be secreted into the intercellular space and thus are not recognized by PRRs in the plasma membrane (Fang et al., 2016). However, our result showed that AGLIP1 was located at ER when expressed in rice protoplasts and *N. benthamiana* (Figure 3B and Supplementary Figure S3), indicating that the protein secreted by *R. solani* may have multiple functions in plant. Alternatively, it is also possible that this effector protein was translocated into the cell after secretion and recognized by cytoplasmic receptors to trigger cell death.

Pathogen effectors often inhibit PTI during compatible interactions, thus enhancing pathogenesis. AGLIP1 was considered as a putative effector because *AGLIP1* was up-regulated during *R. solani* infecting to rice sheaths (Figure 3A), which is a common characteristic of filamentous fungal pathogens effector proteins (Fang et al., 2016). Ectopic expression of pathogen effectors in host plants has been widely used to investigate the virulence of bacterial and fungal effectors (Li et al., 2015; Fang et al., 2019). Subsequently, we demonstrated that immune responses, including *PR* genes expression, induced by flg22 and chitin were dramatically suppressed when AGLIP1 expression was induced in transgenic *Arabidopsis* seedlings (Figure 4). Furthermore, the AGLIP1-expressing transgenic *Arabidopsis* plants showed almost complete inhibition of defense genes expression triggered by the *Pst* DC3000 *hrcC* mutant. Most importantly, ectopic expression of AGLIP1 in the transgenic plants accelerated bacterial colonization and multiplication *in planta* and facilitated disease progression (Figure 5). Similar results have shown that *PR* genes expression induced by *X. campestris* pv. *campestris hrcC* mutant are significantly inhibited in the *X. oryzae* pv. *oryzae* effector XopR expressed in *Arabidopsis* (Akimoto-Tomiyama et al., 2012). The phenomenon that transgenic plants are more susceptible to *Pst* DC3000 *hrcC* mutant than in the wild-type counterpart when AGLIP1 is expressed, indicating AGLIP1 is a critical virulence factor in *R. solani*.

AGLIP1 is likely cytotoxic to rice and *N. benthamiana* but surprisingly displays no toxicity in *Arabidopsis*. It is possible that the DEX-induced expression of AGLIP1 in transgenic lines of *Arabidopsis* suppresses immunity at early infection stages when the transcripts are low, while AGLIP1 promotes cell death at the later stages when transcripts accumulate. The kinetics of the AGLIP1 expression in Figure 3A is further supportive to this notion. Alternatively, AGLIP1 may induce cell death via targeting a specific protein in rice and *N. benthamiana*; such interaction results in decreased accumulation of the targeted protein, which triggers plant cell death. In other words, the function of AGLIP1 may depend on the host. A similar function is found in SsSSVP1 secreted by *S. sclerotiorum* (Lyu et al., 2016). Furthermore, there are other effectors that display cytotoxic activity but also have additional functions. The core effector NIS1 in *Colletotrichum* spp. triggers the cell death of *N. benthamiana* and soybean, and suppresses PAMP-triggered immunity via targeting plant immune kinases (Yoshino et al., 2012; Irieda et al., 2019). The necrosis- and ethylene-inducing protein 1 (Nep1)-like proteins (NLPs) have both cytotoxic and non-cytotoxic functions to different plants (Seidl and Van den Ackerveken, 2019). Therefore, the precise function of AGLIP1 in plant cells needs to be further explored.

Taken together, the findings in this study further deepen our understanding of the effector function in plant pathogenesis of the necrotrophic fungus *R. solani*, highlighting the necessity of large-scale screening and functional analysis of candidate effectors in necrotrophic pathogen with a wide range of hosts. The exact molecular mechanism of how AGLIP1 regulate the rice-*R. solani* interaction remains to be further investigated.

## Materials and Methods

### Bacterial Strains, Plant Materials, and Growth Conditions

The virulent *R. solani* researched in this study were isolated from a heavily infected rice plant in Liaoning province and cultured in PDA medium (200 g potato infusion, 20 g dextrose and 20 g agar*ı* per liter). *N. benthamiana* plants were grown in growth chambers under 14 h/10 h photoperiod and kept at 25°C and 23°C at daytime and nighttime, respectively. *Arabidopsis* plants were grown under 12 h/12 h photoperiod and were kept at 23°C at daytime and 22°C at nighttime, respectively. *A. tumefaciens* EHA105 and GV3101 were cultured in LB medium (10 g tryptone, 10 g NaCl and 5 g yeast extract per liter). *Pst* DC3000 *hrcC* mutant were cultured in KB medium (2% proteose peptone, 0.2% K_2_HPO_4_⋅3H_2_O, 0.15% MgSO_4_⋅7H_2_O, 1% glycerol). The concentrations of antibiotics used in this study are: ampicillin, 100 μg/ml; kanamycin, 50 μg/ml; and rifampin, 25 μg/ml. All data is based on at least three times repeats with similar results.

### Plasmid Construction of *R. solani* Putative Effector Genes

*Rhizoctonia solani* total RNA extraction was based on the manufacturer’s instructions of RNA extraction kit (TaKaRa). Complementary DNA synthetization was performed by using PrimeScript^TM^ 1st Strand cDNA Synthesis Kit (TaKaRa). Phanta Max Super-Fidelity DNA Polymerase (Vazyme) was used for full-length and truncated putative effector-encoding genes amplification. PCR products were digested with *Xho*I and *Spe*I and subcloned into pTA7001 (Aoyama and Chua, 1997), which was constructed with 3 × HA. All constructs were confirmed with sequencing. Primers used in this study are listed in Supplementary Table S2.

### Site-Directed Mutagenesis

Site-directed mutagenesis was performed by splicing overlap extension (SOE) PCR (Li et al., 2015). Two DNA fragments of each effector gene were amplified from the pTA7001-3 × HA gene constructs, respectively. Fusion PCR reaction was performed to combine DNA fragments containing the open reading frame (ORF). The resultant PCR products were cloned into pTA7001-3 × HA after *Xho*I and *Spe*I digestion.

### Transient Expression of Effector Proteins in *N. benthamiana*

Using the freeze-thaw method, the constructed plasmids were transformed into the *Agrobacterium* spp. strain EHA105 (Deblaere et al., 1985). *Agrobacterium* strains were collected and resuspended in MMA buffer (10 mM MES, pH 5.7, 10 mM MgCl_2_, and 150 μM acetosyringone) to an optical cell density of 0.3 at 600 nm after overnight culture, then perform infiltration with needleless syringes after incubation for 3–6 h. All leaves were sprayed with 30 μM DEX at 24 h after infiltration. Leaves within 3 days post DEX spraying were observed and photographed the cell-death phenotypes.

### Rice Protoplast Transfection, Luminescence Measurement, and Subcellular Localization

Rice protoplast isolation and transfection were carried out as described previously (Wang et al., 2018). Briefly, protoplasts were extracted from *Oryza sativa* cv. Nipponbare etiolated seedlings and then transfected with plasmid DNA by polyethylene glycol-mediated transfection. Upon washing with W5 solution, the protoplasts were incubated in W5 solution and under low light for 12 h.

Extracted proteins (20 μl) from protoplast were used for luminescence (LUC) activity detection after mixing with the substrate luciferin (1 mM, 20 μl) and 100 μl of Tricine buffer (20 mM Tricine, 27 mM MgSO_4_⋅7H_2_O, 0.1 mM EDTA, 2 mM DTT, 5 μM ATP, pH 7.8) as described previously (Fang et al., 2016). A microplate reader was used for data determination.

For subcellular localization, the coding sequence *AGLIP1* was amplified and introduced into pUC19-35S-GFP after digestion with *Bam*HI and *Sal*I (Li et al., 2015). The construct was confirmed by sequencing. Transfected rice protoplasts with GFP and RFP fluorescence were observed using confocal microscopy (Olympus FV3000).

### *R. solani* Inoculation

Inoculation of the *R. solani* isolate into rice sheaths of *Oryza sativa* cv. Nipponbare was performed as previously described (Zhang et al., 2017). The inoculated sheaths samples were collected at 0, 12, 24, 48, 72, and 96 h post-inoculation, after immediately liquid nitrogen frozen treatment, then kept in −80°C ultra-low temperature refrigerator for further RNA isolation.

### Development of the *AGLIP1* Transgenic *Arabidopsis* Plants

Agrobacterium-mediated floral dipping transformation which described previously was used for the *AGLIP1* transgenetic *Arabidopsis* seedlings generation (Liu L. et al., 2016). Half-strength Murashige and Skoog (MS) medium with 25 μg/mL hygromycin was used for transgenic seedlings screening.

### Plant Inoculation and Bacterial Growth Assays

*In planta* bacterial inoculation and population sizes were analyzed as previously described (Liu L. et al., 2016). The 4–5 weeks old *Arabidopsis* plants were treated with 30 μM DEX or mock solution before bacterial inoculation after 24 h. Bacterial cells were collected and resuspended in 10 mM MgCl_2_ to OD600 = 0.0005 after overnight culture. Bacterial inoculation was performed by pressure infiltration via plastic needleless syringes. The inoculated plants were covered with plastic sheets to maintain high humidity for 1 day, and then transported to normal growth conditions.

### RNA Extraction and Quantitative Real Time RT-PCR

Samples from seedlings or plants were collected at different periods after *Arabidopsis* seedlings (10 days old) were treated with 1 mM flg22 or chitin or mock solution. Alternatively, 4–5 weeks old *Arabidopsis* plants were spray-inoculated with *Pst* DC3000 *hrcC* mutant at OD600 = 0.2. Total RNA isolation and cDNA was synthesized and performed according to the method described above.

Quantitative real time qRT-PCR was performed according to the manufacturer’s instructions of Bio-Red CFX96 sequence detection system and using ChamQ SYBR Color qPCR Master Mix from Vazyme Biotech Co., Ltd. The expression level of *AtUBQ5* and *gpd* were used as an internal reference for *Arabidopsis* and *R. solani*, respectively. The primer sets used for qRT-PCR are listed in Supplementary Table S2.

### Protein Extraction and Immunoblotting

Samples from *N. benthamiana* leaves which were infiltrated with *Agrobacterium* or from the *AGLIP1* transgenic *Arabidopsis* seedlings were harvested at 24 h after DEX or mock (0.03% ethanol) treatment and were frozen in liquid nitrogen, then grounded in centrifuge tubes with small stainless steel balls by utilizing a milling apparatus (Retsch, Haan, Germany) for total protein extraction. The powders were incubated with 1 × sodium dodecyl sulfate (SDS)-polyacrylamide gel electrophoresis sample buffer (50 mM Tris–HCl, pH 7.4, 2% sodium dodecyl sulfate, 6% glycerol, 0.1 M dithiothreitol, and 0.01% bromophenol blue) and boiled for 10 min.

The extracted proteins were separated in a 12% polyacrylamide gel and electrophoretically transferred onto Immun-Blot PVDF Membrane (Millipore, Bedford, MA, United States) as described previously (Li et al., 2015).

## Data Availability Statement

All datasets generated for this study are included in the manuscript/Supplementary Files.

## Author Contributions

SL, WS, and SW designed and conceived the project, and wrote the manuscript with contributions of all other authors. SL, XP, YW, KH, FX, YZ, and WL performed the experiments and analyzed the data. All authors read and approved the final version of the manuscript for publication.

## Conflict of Interest

The authors declare that the research was conducted in the absence of any commercial or financial relationships that could be construed as a potential conflict of interest.

## Supplementary Material

FIGURE S1 The two ETI marker genes, **(A)** *NbPR1*, and **(B)** *NbHsr203J*, were not induced after DEX-induced expression of AGLIP1. Leaf tissue was collected from the inoculated sites after 1 day induced-expression of AGLIP1 and control protein GFP in *N. benthamiana*. Expression analyses of *NbPR1* and *NbHsr203J* were analyzed by qRT-PCR assay. Data are means ± standard error (SE). The expression level of *NbActin* was used as an internal reference for normalizing within the samples.

FIGURE S2 Analysis conversation and similarity of AGIP1 with known plant fungal pathogens proteins. **(A)** The predicted domain structure of AGLIP1. SP, signal peptide; lipase domain including Asp105, Ser107, Lys108, Pro111, Asp117, Ser174, and Asp230, were predicted key residues necessary for the lipase activity. **(B)** The evolutionary relationship of AGLIP1 and its homologs from other fungi was inferred using the Neighbor-Joining method. The optimal tree with the sum of branch length = 6.66810195 is shown. The percentage of replicate trees in which the associated taxa clustered together in the bootstrap test (1000 replicates) is shown next to the branches. The tree is drawn to scale, with branch lengths in the same units as those of the evolutionary distances used to infer the phylogenetic tree. The evolutionary distances were computed using the Poisson correction method and are in the units of the number of amino acid substitutions per site. The analysis involved 18 amino acid sequences. All positions containing gaps and missing data were eliminated. There were a total of 157 positions in the final dataset. Evolutionary analyses were conducted in MEGA software. **(C)** Conserved amino acid residues of lipase have been showed. Asterisks (^∗^) indicate the predicted lipase active sites of AGLIP1 which play an important role in inducing cell death.

FIGURE S3 Subcellular localization of AGLIP1-GFP transiently expressed in *N. benthamiana*. The vector pCAMBIA1301 carrying GFP was used as a control. The overlapped fluorescence was observed in *N. benthamiana* when co-expressed with AGLIP1-GFP and HDEL-mCherry. The photo was taken under confocal microscopy before cell death symptom was visible.

FIGURE S4 Induced expression level of AGLIP1 in the T2 transgenic homozygous lines Line 1 to Line 7 after DEX or mock treatment. The AGLIP1-3 × HA fusion was detected by Western blotting with an anti-HA antibody. DEX, dexamethasone; Mock, 0.03% ethanol. Each sample was harvested at 24 h after 10 μM DEX or mock treatment. The expression level of *AtUBQ5* was used as an internal reference for normalizing within the samples. CBB (coomassie brilliant blue) staining shows the equal loading of the total proteins. “M” means premixed protein marker.

TABLE S1 Putative effector genes in *Rhizoctonia solani* AG1 IA used for testing cell death-inducing ability.

TABLE S2 The designed primers used in this study.
