# Supplementary figures and images for: The Effector AGLIP1 in Rhizoctonia solani AG1 IA Triggers Cell Death in Plants and Promotes Disease Development Through Inhibiting PAMP-Triggered Immunity in Arabidopsis thaliana
Source: Front Microbiol. 2019 Sep 26;10:2228. doi: 10.3389/fmicb.2019.02228 (PMC6775501; doi:10.3389/fmicb.2019.02228)

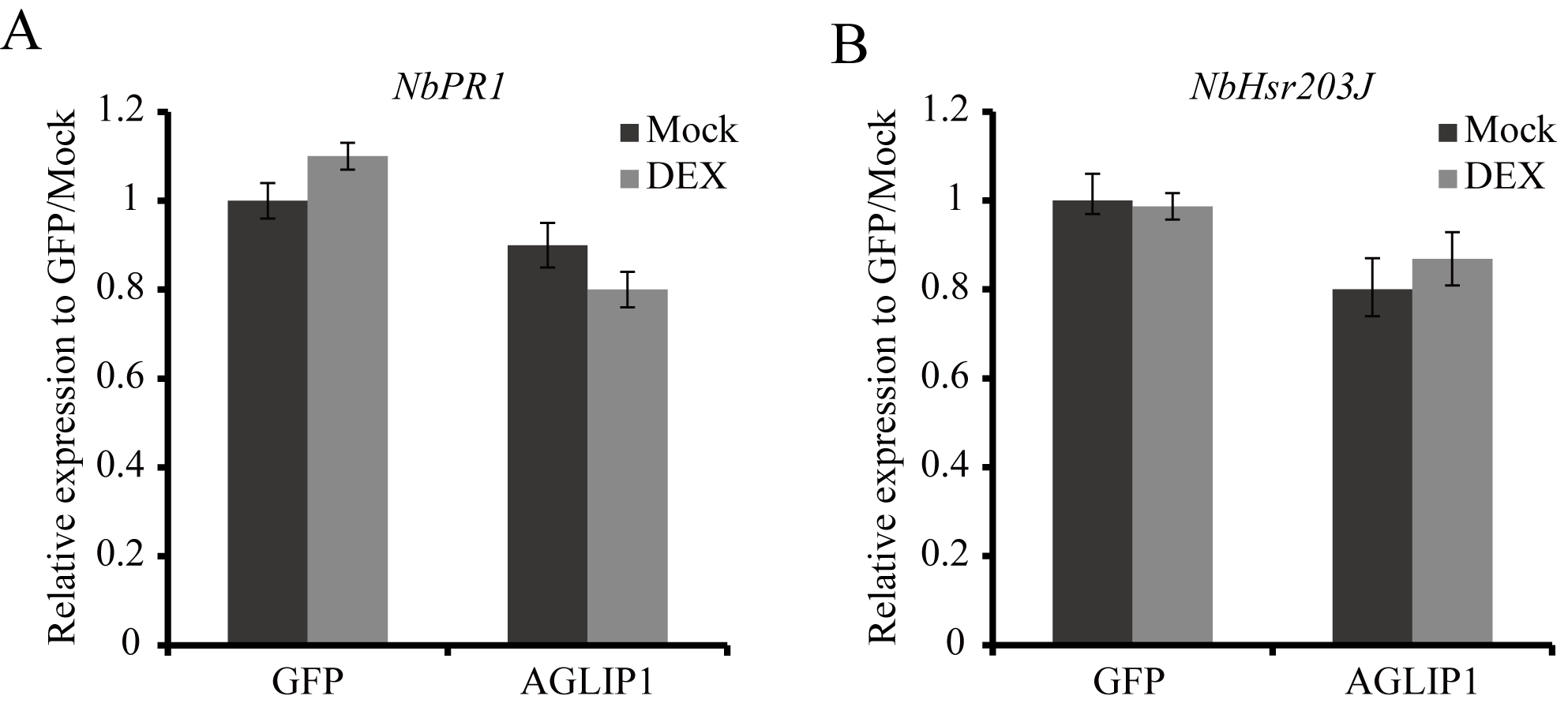

Supplement: FIGURE S1 — The two ETI marker genes, (A) NbPR1, and (B) NbHsr203J, were not induced after DEX-induced expression of AGLIP1. Leaf tissue was collected from the inoculated sites after 1 day induced-expression of AGLIP1 and control protein GFP in N. benthamiana. Expression analyses of NbPR1 and NbHsr203J were analyzed by qRT-PCR assay. Data are means ± standard error (SE). The expression level of NbActin was used as an internal reference for normalizing within the samples. [file Image_1.TIF]

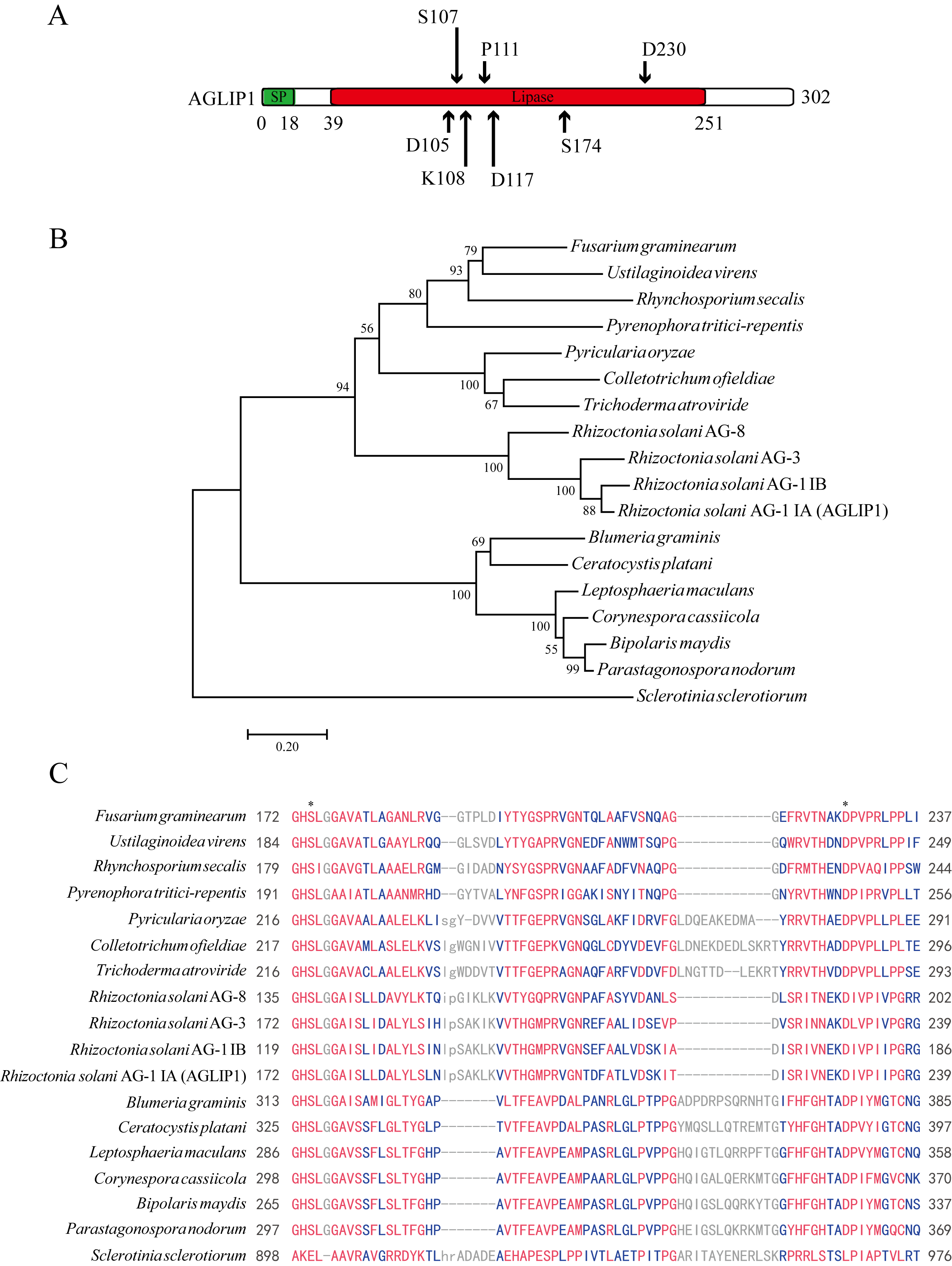

Supplement: FIGURE S2 — Analysis conversation and similarity of AGIP1 with known plant fungal pathogens proteins. (A) The predicted domain structure of AGLIP1. SP, signal peptide; lipase domain including Asp105, Ser107, Lys108, Pro111, Asp117, Ser174, and Asp230, were predicted key residues necessary for the lipase activity. (B) The evolutionary relationship of AGLIP1 and its homologs from other fungi was inferred using the Neighbor-Joining method. The optimal tree with the sum of branch length = 6.66810195 is shown. The percentage of replicate trees in which the associated taxa clustered together in the bootstrap test (1000 replicates) is shown next to the branches. The tree is drawn to scale, with branch lengths in the same units as those of the evolutionary distances used to infer the phylogenetic tree. The evolutionary distances were computed using the Poisson correction method and are in the units of the number of amino acid substitutions per site. The analysis involved 18 amino acid sequences. All positions containing gaps and missing data were eliminated. There were a total of 157 positions in the final dataset. Evolutionary analyses were conducted in MEGA software. (C) Conserved amino acid residues of lipase have been showed. Asterisks (∗) indicate the predicted lipase active sites of AGLIP1 which play an important role in inducing cell death. [file Image_2.TIF]

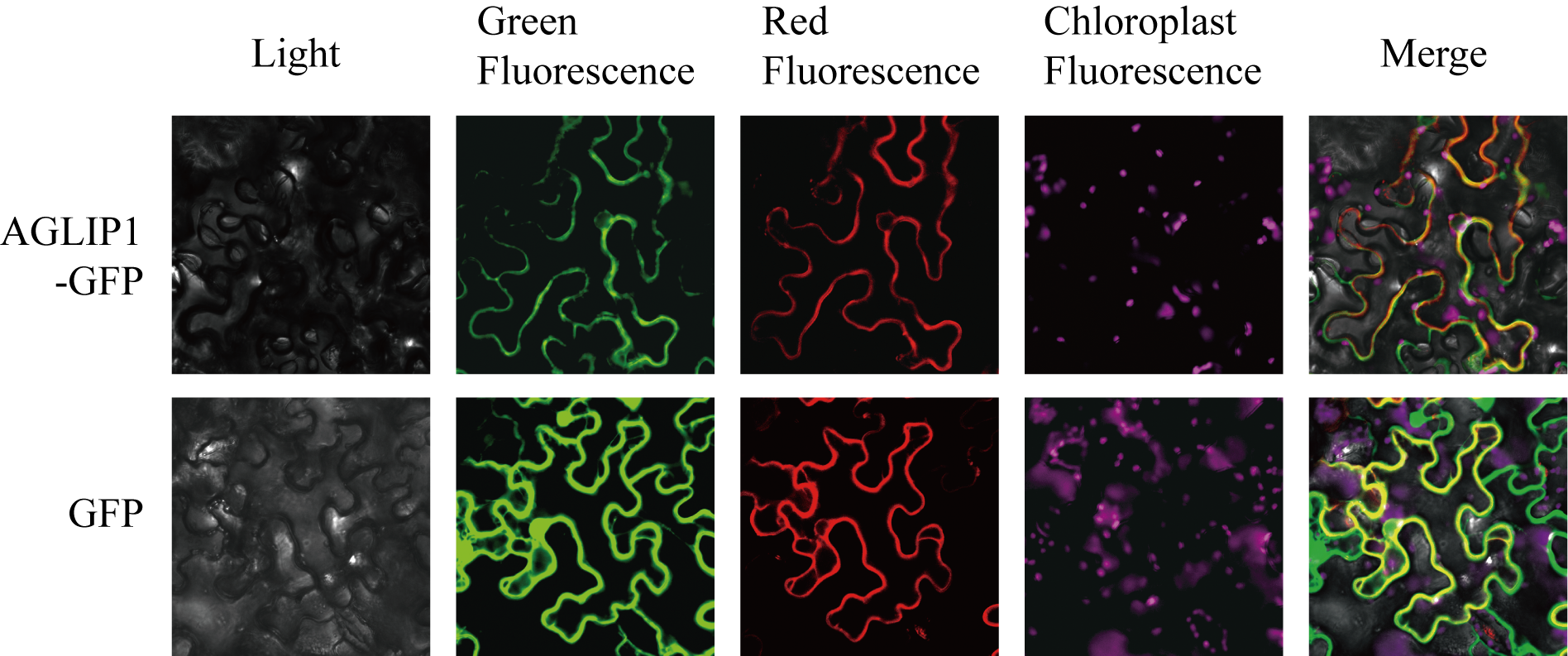

Supplement: FIGURE S3 — Subcellular localization of AGLIP1-GFP transiently expressed in N. benthamiana. The vector pCAMBIA1301 carrying GFP was used as a control. The overlapped fluorescence was observed in N. benthamiana when co-expressed with AGLIP1-GFP and HDEL-mCherry. The photo was taken under confocal microscopy before cell death symptom was visible. [file Image_3.TIF]

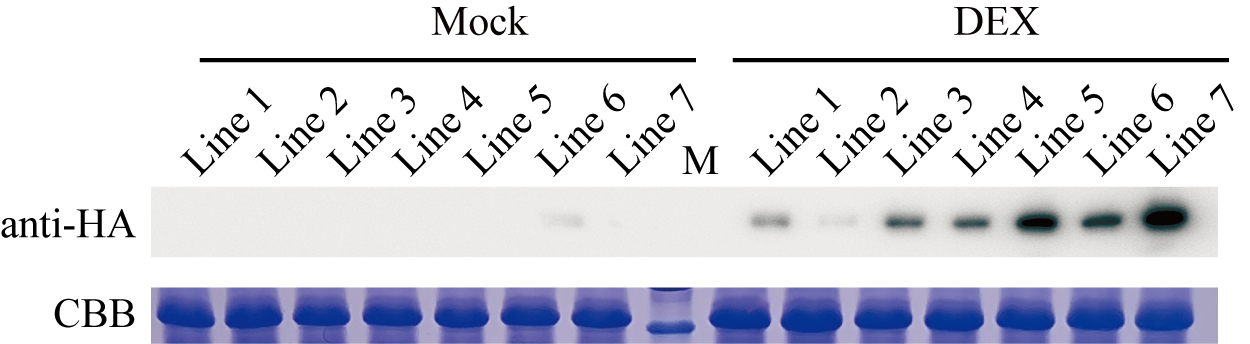

Supplement: FIGURE S4 — Induced expression level of AGLIP1 in the T2 transgenic homozygous lines Line 1 to Line 7 after DEX or mock treatment. The AGLIP1-3 × HA fusion was detected by Western blotting with an anti-HA antibody. DEX, dexamethasone; Mock, 0.03% ethanol. Each sample was harvested at 24 h after 10 μM DEX or mock treatment. The expression level of AtUBQ5 was used as an internal reference for normalizing within the samples. CBB (coomassie brilliant blue) staining shows the equal loading of the total proteins. “M” means premixed protein marker. [file Image_4.TIF]
