# Supplementary material for: The Effector AGLIP1 in Rhizoctonia solani AG1 IA Triggers Cell Death in Plants and Promotes Disease Development Through Inhibiting PAMP-Triggered Immunity in Arabidopsis thaliana
Source: Front Microbiol. 2019 Sep 26;10:2228. doi: 10.3389/fmicb.2019.02228 (PMC6775501; doi:10.3389/fmicb.2019.02228)
Supplement: TABLE S1 — Putative effector genes in Rhizoctonia solani AG1 IA used for testing cell death-inducing ability. [file Table_1.DOCX]

**Table S1.** Putative effector genes in *Rhizoctonia solani* AG1 IA used for testing cell death-inducing ability

| Genes ID | Predicted function (s) | Predicted signal peptide sequence |
| --- | --- | --- |
| AG1IA_05142 (AGLIP1) | Lipase | MLASFAAAFLLGVASTLA |
| AG1IA_08777 | Plastocyanin-like domain-containing protein | MFFNFASIASAAILALPLVGVAA |
| AG1IA_08487 | Ribonuclease domain-containing protein | MYTLLQTVVVVALSGLALA |
| AG1IA_00157 | Polysaccharide deacetylase domain-containing protein | MHFAVITATLFGVVGVSA |
| AG1IA_09049 | Endoplasmic reticulum metallopeptidase | MTKSTSVWHTFASLLPLLVITPWITS |
| AG1IA_07285 | Peptidase inhibitor i9 domain-containing protein | MRSTFILACLASSVCAVFA |
| AG1IA_09356 | Polysaccharide lyase family 1 protein | MKFTASALALITGLGVVSANPLL |
| AG1IA_00669 | Glycosyl hydrolase family 61 domain-containing protein | MLFSSLVSFVALAAAGVNA |
| AG1IA_09650 | Multicopper oxidase domain-containing protein | MRISTLFPTVALLWVALVDA |
| AG1IA_09940 | 1,4-beta-D-glucan cellobiohydrolase B | MYFLALIPLYLAAANA |
| AG1IA_03106 | Glycoside hydrolase family protein | MNIICRSAIVLFLSSTAIA |
| AG1IA_03129 | GDP-mannose 4,6-dehydratase | MAAPIAPAAFALSRHG |
| AG1IA_03694 | Aspartic protease | MLPFIAVSALVATQSALA |
