# Supplementary material for: The Effector AGLIP1 in Rhizoctonia solani AG1 IA Triggers Cell Death in Plants and Promotes Disease Development Through Inhibiting PAMP-Triggered Immunity in Arabidopsis thaliana
Source: Front Microbiol. 2019 Sep 26;10:2228. doi: 10.3389/fmicb.2019.02228 (PMC6775501; doi:10.3389/fmicb.2019.02228)
Supplement: TABLE S2 — The designed primers used in this study. [file Table_2.DOCX]

**Supplementary Table S2. The designed primers used in this study**

| Purpose of use | Primer name | DNA sequence |
| --- | --- | --- |
| Cloning | AGLIP1-pTA7001-3HA-F | GACTCTAGCCTCGAGATGCTCGCAAGCTTTGC |
|  | AGLIP1-pTA7001-3HA-R | AATTAACCCACTAGTGTTATTGCAAGCATCGGAAC |
|  | AGLIP1-pUC19-35S-GFP-F | CGGTACCCGGGGATCCATGCTCGCAAGCTTTGCTGC |
|  | AGLIP1-pUC19-35S-GFP -R | TGCTCACCATGCATGCGTTATTGCAAGCATCGGAAC |
|  | 08777-pTA7001-3HA-F | GACTCTAGCCTCGAGATGTTCTTCAACTTTGCTTC |
|  | 08777-pTA7001-3HA-R | AATTAACCCACTAGTCTTTCCTCCTGATTCCTTG |
|  | 08487-pTA7001-3HA-F | GACTCTAGCCTCGAGATGTACACCCTCCTTCAAAC |
|  | 08487-pTA7001-3HA-R | AATTAACCCACTAGTGCGCCGTAGTGAGTAATAAC |
|  | 00157-pTA7001-3HA-F | GACTCTAGCCTCGAGATGCATTTTGCCGTTATTAC |
|  | 00157-pTA7001-3HA-R | AATTAACCCACTAGTAAGCGGGGTATCCTTTGA |
|  | 09049-pTA7001-3HA-F | GACTCTAGCCTCGAGATGACCAAGTCCACGTC |
|  | 09049-pTA7001-3HA-R | AATTAACCCACTAGTCTCGGACATGATAACCC |
|  | 07285-pTA7001-3HA-F | GACTCTAGCCTCGAGATGCGCTCCACATTCATCCTCGCA |
|  | 07285-pTA7001-3HA-R | AATTAACCCACTAGTAGCAGCCGCCACCCGG |
|  | 09356-pTA7001-3HA-F | GACTCTAGCCTCGAGATGAAGTTCACGGCATCTG |
|  | 09356-pTA7001-3HA-R | AATTAACCCACTAGTGAACGAGAGCTTGGCACC |
|  | 00669-pTA7001-3HA-F | GACTCTAGCCTCGAGATGTTGTTCTCTTCGCTTGTA |
|  | 00669-pTA7001-3HA-R | AATTAACCCACTAGTTTCGCCAGTAGAAGTAGGCT |
|  | 09650-pTA7001-3HA-F | GACTCTAGCCTCGAGATGCGTATATCTACCTTGTT |
|  | 09650-pTA7001-3HA-R | AATTAACCCACTAGTCACAATATCAGGCTTCGAGT |
|  | 09940-pTA7001-3HA-F | GACTCTAGCCTCGAGATGTATTTCCTCGCTCTTATCC |
|  | 09940-pTA7001-3HA-R | AATTAACCCACTAGTCTTCTTCTTGGTGTCCACGA |
|  | 03106-pTA7001-3HA-F | GACTCTAGCCTCGAGATGAATATTATCTGTAGATCTGCG |
|  | 03106-pTA7001-3HA-R | AATTAACCCACTAGTCGCCCCAACTCCCAAC |
|  | 03129-pTA7001-3HA-F | GACTCTAGCCTCGAGATGGCTGCTCCTATTGCTC |
|  | 03129-pTA7001-3HA-R | AATTAACCCACTAGTATTTTGATCCTCAACGAGGT |
|  | 03694-pTA7001-3HA-F | GACTCTAGCCTCGAGATGCTTCCTTTTATTGCTGTC |
|  | 03694-pTA7001-3HA-R | AATTAACCCACTAGTAACTGGGGTAGCAAATCCAA |
|  | AvrBs2-pTA7001-3HA-F | GACTCTAGCCTCGAGATGCGTATAGGTCCTCCGCA |
|  | AvrBs2-pTA7001-3HA-R | AATTAACCCACTAGTCTCCGGCTCGGTCTG |
|  | GFP-pTA7001-3HA-F | GACTCTAGCCTCGAGATGGTGAGCAAGGGCGAGGA |
|  | GFP-pTA7001-3HA-R | AATTAACCCACTAGTCTTGTACAGCTCGTCCATGC |
| The mutant construction | D105A-pTA7001-3HA-F | AGGAACCGCTCCATCC |
|  | D105A-pTA7001-3HA-R | GGATGGAGCGGTTCCT |
|  | S107A-pTA7001-3HA-F | GGAACCGATCCAGCCAA |
|  | S107A-pTA7001-3HA-R | TTGGCTGGATCGGTTCC |
|  | K108A-pTA7001-3HA-F | GAACCGATCCATCCGCATT |
|  | K108A-pTA7001-3HA-R | AATGCGGATGGATCGGTTC |
|  | P111A-pTA7001-3HA-F | TTGTGGCACTCTTGATCG |
|  | P111A-pTA7001-3HA-R | CGATCAAGAGTGCCACAA |
|  | D117A-pTA7001-3HA-F | TGATCGATGCCGCCTT |
|  | D117A-pTA7001-3HA-R | AAGGCGGCATCGATCA |
|  | S174A-pTA7001-3HA-F | AGGACACGCTCTCGGT |
|  | S174A-pTA7001-3HA-R | ACCGAGAGCGTGTCCT |
|  | D230A-pTA7001-3HA-F | TCAACGAAAAGGCTATTGTTCC |
|  | D230A-pTA7001-3HA-R | GGAACAATAGCCTTTTCGTTGA |
|  | NSP-pTA7001-3HA-F | GACTCTAGCCTCGAGATGGCTCCCATCCCTCGC |
| Gene expression | FRK1-qPCR-F | GGACACCGCGTATAGTGAGTT |
|  | FRK1-qPCR-R | TGACAGTAGAAGCCGGTTGG |
|  | At1g51890-qPCR-F | TGGACCTCTGTTTCGATCCC |
|  | At1g51890-qPCR-R | GCGACCACAATAAGCGATCC |
|  | At2g17740-qPCR-F | CATGCGGTGAGTATGGATCG |
|  | At2g17740-qPCR-R | TCCTTTGGGCATGGAGCCTT |
|  | At5g57220-qPCR-F | GCCCGAGAAGTTTATGCCTGA |
|  | At5g57220-qPCR-R | TCGTCCGTTACCAAACACCAT |
|  | AGLIP1-qPCR-F | ACCAAGTTTTTCCCCGGTGT |
|  | AGLIP1-qPCR -R | GGTATTTCCAACCCTCGGCA |
|  | NbPR1-qPCR-F | TTGCCGTGCCCAAAATTCTC |
|  | NbPR1-qPCR -R | CTGGTCGTCCCAGGTCAAAG |
|  | NbHsr203J-qPCR-F | AGACGGTTCAGTAGACCGGA |
|  | NbHsr203J-qPCR -R | CGCCGTCGATGAAGTAGTCA |
|  | gpd-qPCR-F | TACTCCGCAATGCTATCG |
|  | gpd-qPCR-R | TACTCGGTCCCAGTGGT |
|  | AtUBQ5-qPCR-F | ACGGGGAAGACCATAACCCT |
|  | AtUBQ5-qPCR-R | GGCGAAAATCAATCGCTGCT |
|  | NbActin-qPCR-F | ACAGGAATGGTCAAGGCTGG |
|  | NbActin-qPCR-R | GCCGGCCAACAATACTAGGG |
